# Systematic review on what works, what does not work and why of implementation of mobile health (mHealth) projects in Africa

**DOI:** 10.1186/1471-2458-14-188

**Published:** 2014-02-21

**Authors:** Clara B Aranda-Jan, Neo Mohutsiwa-Dibe, Svetla Loukanova

**Affiliations:** 1 Department of Engineering, Institute for Manufacturing, 17 Charles Babbage Road, Cambridge CB3 0FS, United Kingdom; 2 Institute of Public Health, University of Heidelberg, Im Neuenheimer Feld 324, 69120 Heidelberg, Germany

**Keywords:** mHealth, Telemedicine, Access to healthcare services, Africa

## Abstract

**Background:**

Access to mobile phone technology has rapidly expanded in developing countries. In Africa, mHealth is a relatively new concept and questions arise regarding reliability of the technology used for health outcomes. This review documents strengths, weaknesses, opportunities, and threats (SWOT) of mHealth projects in Africa.

**Methods:**

A systematic review of peer-reviewed literature on mHealth projects in Africa, between 2003 and 2013, was carried out using PubMed and OvidSP. Data was synthesized using a SWOT analysis methodology. Results were grouped to assess specific aspects of project implementation in terms of sustainability and mid/long-term results, integration to the health system, management process, scale-up and replication, and legal issues, regulations and standards.

**Results:**

Forty-four studies on mHealth projects in Africa were included and classified as: “patient follow-up and medication adherence” (n = 19), “staff training, support and motivation” (n = 2), “staff evaluation, monitoring and guidelines compliance” (n = 4), “drug supply-chain and stock management” (n = 2), “patient education and awareness” (n = 1), “disease surveillance and intervention monitoring” (n = 4), “data collection/transfer and reporting” (n = 10) and “overview of mHealth projects” (n = 2). In general, mHealth projects demonstrate positive health-related outcomes and their success is based on the accessibility, acceptance and low-cost of the technology, effective adaptation to local contexts, strong stakeholder collaboration, and government involvement. Threats such as dependency on funding, unclear healthcare system responsibilities, unreliable infrastructure and lack of evidence on cost-effectiveness challenge their implementation. mHealth projects can potentially be scaled-up to help tackle problems faced by healthcare systems like poor management of drug stocks, weak surveillance and reporting systems or lack of resources.

**Conclusions:**

mHealth in Africa is an innovative approach to delivering health services. In this fast-growing technological field, research opportunities include assessing implications of scaling-up mHealth projects, evaluating cost-effectiveness and impacts on the overall health system.

## Background

According to the International Telecommunication Union (ITU), mobile-phone subscriptions reached almost 6 billion globally in 2011, driven mainly by an increase of subscribers from developing countries which added more than 80% of the 660 million new subscriptions during that year [1]. By 2013, global penetration of mobile phones is estimated to reach over 95% of the population worldwide [2], which means that more people will have access to mobile phones than to water and sanitation services [3]. This shows the large and rapid expansion of mobile phone ownership in the developing world.

Mobile health (mHealth) is a component of electronic health (eHealth). Though not a standardized definition, mHealth refers to the use of mobile communication technologies to promote health by supporting healthcare practices (e.g. health data collection, delivery of healthcare information, or patient observation and provision of care) [4,5]. This technology has erupted rapidly and, consequently, the benefits and limitations for healthcare are still not well understood.

In developing countries, decreasing costs and increasing network coverage provide a wide range of opportunities for applications using mobile phones and other telecommunication technologies. These opportunities can also be extended to the utilization mHealth technologies in healthcare [6]. The use of mHealth services can have the potential to improve affordability of interventions for health promotion, increase health education and disease prevention [7-10]. Mitchell et al. [11] suggest that portability, the “always on” status, and data transmission are the qualities of mobile phones that have made them reach a larger population than computers and the internet. Moreover, telecommunication technologies may also reduce time, distance and cost of information delivery, and support health providers to offer cost-effective services [3]. In developing countries, mHealth could offer solutions for healthcare systems challenged by inadequate finances, poor health information systems, scarce resources and limited trained staff, particularly in countries with a rapid-growing number of mobile phone subscriptions [12]. The present study looks at mHealth as the use of mobile phone technology to enable provision of healthcare services in Africa.

The main objective of this study is to analyze the experiences of mHealth implementations in Africa during the last decade, and to identify factors influencing the successes and failures of mHealth projects in Africa using a SWOT (strengths, weaknesses, opportunities and threats) analysis. Our specific research questions are:

a) What are the factors leading to successful implementation of mHealth projects?

b) What are the factors limiting or challenging the implementation of mHealth projects?

c) Why do these factors cause project failure or limit project implementation?

Through answering these research questions we aim to gain a better understanding of the current situation of mHealth projects in Africa, develop recommendations based on these findings and identify areas were further research is needed.

### Significance of this study

mHealth is an emerging topic and most projects have recently been implemented, or are in a pilot stage. Therefore, their duration is too short to be able to accurately measure their impacts [8]. The absence of such information may hinder efforts to understand limitations, challenges and reasons for success of mHealth projects. By documenting and assessing experiences, we aim to inform on the issues faced during mHealth project implementation.

## Methods

### Literature search strategy and criteria for study selection

An electronic systematic literature search was conducted using PubMed and Journal @Ovid. Two search strategies were used: the first one combined the MESH terms “mHealth” AND “Africa”, and the second search combined the free-text words “mobile phone$ or cellphone$” AND “health” AND “Africa”. The searches were limited to articles published in English during the period between 2003-2013. The searches were performed in June 2013 by two authors (CAJ and SL). All duplicated articles were removed automatically using Endnote and a manual revision was done for verification (CAJ). From the total search results, all potential abstracts were screened and studies were selected for full-text review (CAJ and NMD). Full-text articles were searched manually in digital sources and studies were excluded when access to full-text articles was not available. To avoid selection bias, the three authors carried out the full-text article review and any difference in the selection was discussed and papers selected accordingly. Exclusion criteria were: Project not located in Africa, non-mHealth implementation (telemedicine, other types of eHealth and use of other telecommunication technologies, such computers, internet or e-mail), and studies on factors associated to mobile phones but not mHealth implementation (e.g. community ownership or acceptability of mobile phones). Except for project protocols, all study designs (randomized-control trials [RCTs], pilot project, literature reviews etc.) were included.

### Data collection and analysis

Based on the research questions and objectives, data from full text articles was summarized, extracted and collected manually in a table format in four main groups: strengths, weaknesses, opportunities and threats (SWOT). The rationale behind the use of a SWOT analysis is that it allows identifying internal and external factors influencing the performance of a project. This SWOT-type analysis is particularly useful for strategic project planning and has been widely used in management and policy research, as well as being one of the main tools used to inform decision-makers about effectiveness of projects [13]. For these reasons, we considered it to be an adequate tool to strategically assess implementations of mHealth projects. Considering the objectives of the present analysis, each SWOT group refers to:

• Strengths: internal factors referring to outcomes, project drivers, reasons for success.

• Weaknesses: internal factors referring to project limitations and challenges.

• Opportunities: external factors such as areas of potential for mHealth implementation, facilitators of mHealth projects, etc.

• Threats: external factors such as potential for failure, external barriers and limitations.

Articles included after the full-text review were analyzed according to these indicators and findings were compiled in a SWOT table into six different areas of project implementation (Table 1):

• Project sustainability: mid- and long term results and impacts.

• Project integration into the health system: relevance of the design, involvement of key stakeholders, compatibility to existing government policies and management information systems.

• Technology/existing infrastructure: cost, usage and acceptance, network coverage, electricity and other infrastructure.

• Project management process: related resources required for project implementation.

• Scale-up and replication: requirements for scaling-up projects at a regional or national level.

• Legal issues, regulations and standards: in-country regulations, laws or standards that influence mHealth projects.

**Table 1:** SWOT analysis of included studies

**Factors**	**Strengths**	**Weaknesses**	**Opportunities**	**Threats**
**Mid-and long-term results/project sustainability**	**-Improve delivery of services (e.g. skilled delivery attendance) **[65]** and service request (e.g. appointments) **[27]**,**[34]**,**[62]	**-Unclear benefits, uncertain long-term results and effectiveness (e.g. insufficient results from RCTs) **[16]**,**[32]**,**[37]**,**[46]**,**[61]**,**[63]**, and unclear cost-benefit analysis **[29]**,**[61]**.**	-Potential to enhance timeliness in reporting health and stock data in rural and remote areas [34]	-High facility workload and staff/patient/user illiteracy [38,54]
	-Improved patient-health worker and clinic staff-health worker communication [31]	-Results are variable depending on the duration of the intervention and may be overestimated [55], limited study design and external validity [20,21], weak evidences [27]	-Lack of stock management resulting in patients untreated [49]	-Limited knowledge on the effects of mHealth on patient health outcomes in low-resource settings [15,62]
	**-Increased health workers’ adherence to clinical guidelines and quality of treatment **[17]**,**[22]**,**[34]**,**[37]**,** worker morale and sense of empowerment [43]**, access to medical/health information at the point-of-care **[17]**,**[37]**,**[50]**,** and motivation due to training and improved skills [32]	-Difficult to monitor text messages content [23], high possibility of data under-reporting [37,46], and possibility of biased responses from participants [14]	-mHealth projects are regarded as innovative and current data collection methods tend to have poor quality [47]	-Use of mobile technology for research is recent [22]
	-Higher rate and more efficient patient follow-up [33], **uptake of counselling and testing **[22]**,**[31]**,**[50]**,** reporting of adverse reaction to treatment [24], improved patient’s adherence and response to treatment [15], and **higher detection of adherence failure **[21]**,**[22]**,**[30]**-**[32]**,**[37]**.**	-Reported patient anxiety due receiving information [61]		-Dependency in donor funding and limited funding opportunities may limit long-term sustainability [56]
	-Supports efficient stock management, local drug distribution, counting and ordering accuracy, and supply chain monitoring [22]			-mHealth results are dependant of external factors (e.g. long duration of patient treatment may reduce adherence and motivation to participate) [18,63]
	**-Overcome communication delays, ensure real-time data acquisition and reporting, reduces data losses and monitor data quality **[46]**,**[47]**,**[49]**, makes available pre-define indicators and reduces delayed reporting **[14]**,**[18]**,**[38]**,**[46]**,**[50,51]**,**[54]**,**[56]**,**[63]			
	**-Decreases referral time and care costs burden to patients due to transportation **[49]**,**[51]**,**[62]			
	**-Supports disease surveillance systems and monitoring of interventions **[25]**,**[31]**,**[34]			
	-Allows delivery of lab text results [51,57] and reduction of facility’s turnaround time [34,40]			
	-Overcome logistical and distance barriers [40], and reduce operational costs [17,40]			
	-Provide health education [39]			
**Integration into the health system**	-Support patient management [20]	-Unclear roles, responsibilities, actions, boundaries and responses needed at different levels of healthcare system (government) for project implementation and scale-up [45]	-Existing communication gap between health workers, managers and patients [63]	-Political crisis may hindered project implementation and results [26,50]
	-Intervention flexible to be adapted to local context and language [31,62]	-Project results depend on training and clinical practice of health workers [49,63]	-Weak routine health, logistics, and surveillance data reporting systems [62]	-Current care delivery processes will need to be redesigned (e.g. change to electronic records and data) [22]
	-Allows focusing efforts of clinical staff in areas not covered by the intervention (e.g. remote areas with no mobile phone coverage) [37,56]	-Most pilot projects are started by implementing organisations themselves rather than integrated to the health system [45]	-Monitoring and evaluation of programmes may be done with collection of electronic information[62]	-Costs of mHealth implementation may affect patient treatment costs [55]
	-Public-private partnerships proved to work effectively in these projects [25,37]	-mHealth projects are unlikely to prove effective in poorly performing systems [63]	-Improved adherence to clinical guidelines by health workers is required [52]	-Unknown health systems complexities for large scale implementation of mHealth projects [55]
	-High government commitment, existing governmental eHealth strategy [47]		**-Poor management of drug supply chain and large discrepancies of and limited control in stock levels of health facilities **[43]**,** and poor stock forecasting [15,47,49]	-Lack of cultural and organisational capacity to manage digital health information [63] may lead to late reporting, lack of feedback and incomplete data collection [63]
	-Availability of local private providers willing to set up the mHealth system [47]		**-Opportunities to be implemented in different national disease control programmes; provide access to data for an evidence-based approach **[47]**,**[50]	
	-Increased participation of local health staff in active case detection in surveillance systems (e.g. malaria) [49]		**-Project may be attractive and acceptable for private or commercial partners and governments (MoH) **[21]**,**[29]**,**[49]**,**[50]	
	-Places rural health centres in direct communication with the MoH and other stakeholders [45,50]		-Underutilise community health workforce (e.g. health workers) [19,47,49] and lack of specialised care/mentoring in rural areas [26,50]	
			-Difficult to collect and disseminate health data in remote areas [33,58]	
**Project management process**	-Support provision of user and staff training [52]	-Low patient motivation to participate (e.g. reply messages or calls) [54,56], particularly if project is not tailored to their needs (e.g. local language) [32]	-Implementation needs to become multidisciplinary [44]	-Challenge of management of mHealth projects remain underestimated [26]
	-Minimal human resources and training are required [32,54]	-Small sample size of pilot projects provide limited or biased results [20,31]	-Available funding from larger programmes (e.g. PEPFAR mobile clinic) [62]	
	-Financial incentive (e.g. airtime credit) allows high response rate to the project [21,22]	-Costs and logistics affect text messaging responding on time [14,33,61]	-Reporting transparency for donors and stakeholders [37]	
	-Allows real-time supervision and monitoring work rate, attendance, and staff working hours [47,50]	-Occasional staff shortages during project implementation [22], and staff may be overwhelmed of increased calls or messages [50]	-Low capacity and administrative challenges for data collection [49]	
			-Research is needed to optimize project delivery and intervention targets [31]	
**Legal issues, regulations and standards**	-Coded information contributes to data security and confidentiality [63]	-Privacy concerns raised when using mobile phones, particularly if not owned by the patient [34,56]	Not mentioned	-No minimum number of critical surveillance parameters to be reported has been established [34]
	-Integration of SMS guidelines into healthcare process delivery [50]	-Security measures (e.g. PIN) may be confusing to users when unfamiliar and poorly understood [37,54] and expectations are variable for maintaining confidentiality [32]		-Lack of published data on feasibility and acceptability of confidentiality methods [62]
				-Unknown standards for monitoring and evaluation of mHealth programmes [21,34]
**Technology and infrastructure**	**-Text messaging is inexpensive, uses existing infrastructure (e.g. existing networks, reducing phone costs) **[18]**,**[22]**,**[26]**,**[49]**,**[50]**,**[56]**,**[58]**,**[59]**,**[62]**,**[63]**, and is easy to use **[43]	-Limited text capacity of mobile phones and text messages (e.g. up to 160 char.) [18]	**-High access and rapid expansion of mobile network coverage, availability of inexpensive handsets, and decreasing costs of mobile phone services and rapidly-growing technological field **[14]**-**[19,22,26,28,31,34,37,40],[43,49,56,62]	-High phone theft and limited electricity to charge phones [38]
	**-Users are familiar to mobile phone services **[31]**,**[34]**,**[47]**,**[53]**,**[59]**,**[62]	-Staff are not always able to use or act promptly to the text messaging requests, or do not have the skills required [43,62]	-Potential of SMSs to influence uptake of healthcare technologies [33,57]	-Technical or expert knowledge for development, maintenance and platforms (software and hardware) may be limited [37,56], and slowdown implementation [49,57,62]
	**-High acceptance, satisfaction and valued by patients and staff **[16]**,**[18]**,**[33]**,**[34]**,**[47]**,**[49]**,**[56]**,**[63]	-Variable access to mobile phones (e.g. not all patients own a personal phone, phones are often shared, cost of service) [32,37]	-SMS-based software and delivery systems can be updated and review for future developments [24]	**-Dependency on network coverage **[19]
	-Mobile phones are not easily broken and less subject to thief than other technologies [17,22,25,28,34,54,63]	**-Technical challenges reduce data quality and transfer **[18]**,**[37]**, lost network, phone maintenance costs **[17]**and risk of theft and lose **[19]	-The lack of other communication technologies (e.g. internet) offers opportunities to mobile phones [55]	**-High illiteracy and users’ preference makes voice calls more attractive than text messaging **[14]**,**[17]**,**[62]
		-Use of similar technologies may not have similar results [21,25,54]		**-Unreliable network, internet and electricity access **[32]**,**[34]**,**[37]
		-Staff may not use the mobile phones appropriately or handle them with care [57]		-Receptiveness of the technology is limited by socioeconomic and sociocultural factors, geographic barriers and quality of care [19,20,32,33,49]
		-Software may not be adaptable or flexible, and are still subject to human error [63]		
**Scale-up and replication**	-Allows monitoring and impact assessment prior to scaling-up [27]	-No assessment has been performed to know if an effective implementation for one disease works for other diseases [24,40,44,54,62]	**-Cost-effective implementation of m-Health programmes (e.g. lower running costs) **[59]	**-Unknown cost-effectiveness of deployment and maintenance **[56]
	-Feasible to be implemented in remote and resource-limited areas [49], and potential nationwide scale-up [32,56]	-High upfront set-up costs [43], difficulties to secure sustainable funding for scaling-up [19,32], and uncertainty on future changes of costs [63]	-Innovations for automated text messaging and partnerships with mobile technology developers may improve scalability [37,39,49]	- Lack of a mechanism to use data collected at district and national levels [17,32,43,46,56]
	**-Low replication costs and highly adaptable to specific cultural contexts **[51]		-Open source programmes may support implementation of mHealth in low-resource settings [22]	-MoH guidance and policies, and government financial support are lacking and are required for scaling-up [49]
	**-High potential to be scaled-up **[32]**,**[47]**,**[56]			-Little existing evidence on efficacy and effectiveness of mHealth interventions [49,63], particularly at large-scale [32]

The table below summarizes and compiles findings of the studies included in the review. The SWOT analysis methodology was used in five areas related to implementation of mHealth projects: project sustainability and mid/long-term results, integration of the project to the health system, technology and infrastructure, project management process, scale-up and replication, and legal, regulatory and standardization aspects. When more than 3 studies were found to be referring to the same issues, these were **embolden** to highlight their relevance according to appearance in the studies.

## Results and discussion

**Figure 1:**
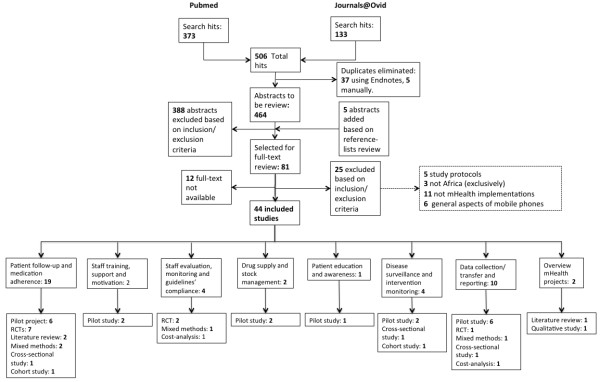
Inclusion/exclusion flowchart.

From a total of 464 search results, 81 studies were selected for full-text review of which 44 studies, published between 2006 and 2013, were included in the review according to the inclusion criteria. These studies include 19 pilot studies, 11 randomized-control trials, 4 mixed methods studies, 3 cross-sectional studies, 2 cohort studies, 1 qualitative study (interviews), 2 literature reviews, and 2 cost-analysis studies. Most of the mHealth projects focused on HIV, malaria, tuberculosis (TB), diabetes and antenatal care. Further screening allowed their classification into topics according to mHealth benefits and types of intervention into: “patient follow-up and medication adherence” (n = 19), “staff training, support and motivation” (n = 2), “staff evaluation, monitoring and guidelines compliance” (n = 4), “drug supply-chain and stock management” (n = 2), “patient education and awareness” (n = 1), “disease surveillance and intervention monitoring” (n = 4), “data collection/transfer and reporting” (n = 10) and “overview of mHealth projects” (N = 2). When a project fell into two or more of these topics, the authors selected the most fitting and classified the project as such. Figure 1 presents a flowchart detailing the inclusion/exclusion process.

Most projects were pilot studies or RCT studies implemented at community levels and not yet scaled-up to larger levels, thus evidence presented on effectiveness is limited and long-term results are unclear. Nonetheless, findings show the feasibility and potential of these projects to support healthcare systems in Africa. The following sections will, firstly, discuss the findings on each topic (except “overview of mHealth projects”, included in the SWOT discussion) and, secondly, present the results of the SWOT analysis of each aspect of project implementation.

### Patient follow-up and medication adherence

Implementations to improve patient life-style and medication adherence, and treatment follow-up were the most common among the findings of the review (n = 19). Six studies were results from pilot projects to inform birth outcomes [14], report drugs’ secondary effects [15], follow-up children’s vaccinations [16], monitor patients with TB [17], observe of diabetic patients’ behavioral change [18] and identify pregnant women needing antenatal care and referral services [19]. RCT studies were also common in this topic (n = 8), showing results on ART monitoring and patient adherence [20-26] and skilled delivery attendance [27]. SMS, video messages (MMS) and phone calls were used on these projects. Other findings included a cross-sectional study exploring the feasibility of using mobile phones for sending reminders to patients regarding their medications and appointments in an antiretroviral treatment (ART) health facility [28], a review to determine effectiveness of SMS on patient adherence to ART [29], a cohort study to monitor patient adherence to ART [30], and two mixed-methods studies, one for supporting patients with breast cancer during their oncological treatment [31] and a second one to monitor adherence to treatment and care provided by caregivers of HIV-infected children [32].

When comparing pilot projects to RCTs, outcomes are not always consistent. Pilot projects tend to report outcomes positively and draw on the high potential for mHealth to be effective in providing solutions needed in the healthcare system. RCTs and mixed-methods studies, on the other hand, differ from these results by reporting little to no significant effect of the mHealth intervention; this is particularly true for projects on patient adherence to ART.

The feasibility and potential of mHealth implementations for patient adherence to treatment and follow-up is unanimously agreed upon across the studies. However, the reproducibility and scalability of these projects is far from certain. The various studies discuss problems such as a lack of adjustment to confounding factors [27], small sample sizes [14,33,34], lack of external validity [27], uncertainty in data quality [14], and lack of larger RCTs in the literature[29] as factors that make projects’ outcomes questionable.

### Staff training, support and motivation

The lack of trained and qualified health staff is a major challenge for many African health systems [34,35]. In Uganda and South Africa, in areas where staff and infrastructure are limited, the use of mobile phone technologies by community health workers (CHW) gave positive results on HIV-infected patient care [36]. Frequent delivery of text messages has positively influenced patient care and logistics, supporting clinic and community health workers and patients [26,37,38].

Two pilot projects were included that aim to support, train or provide motivation to healthcare workers or staff clinicians. A pilot project in Botswana showed that health workers in remote areas could be linked to specialists to get advice for making better diagnoses by accessing point-of-care medical information [33]. The second project trained community health workers to utilize mobile phones for reporting on patient adherence, send reminders for appointments, and answer physician queries [39]. The project showed that by supporting health workers using mobile phones the facility’s operational costs and worker-time were decreased, while capacity of the treatment program increased. These projects show that mHealth could benefit developing countries by accessing clinical information in rural areas and, transferring clinical data and build capacity [40-42].

### Staff evaluation, monitoring and compliance to guidelines

mHealth has also been used for staff evaluation and performance monitoring [37,43,44] and compliance to treatment guidelines [45]. Studies included three RCTs and one cost-analysis. In Kenya and Uganda, the use of SMS reminders helped to improve patient care and had a positive effect in case management [37,45]. In Kenya, studies showed that mHealth is a cost-effective tool for improving quality of treatment and provider performance with limited resources [43,44]. However, similar to patients’ adherence, RCTs show surprising results regarding health workers’ compliance to guidelines. For instance, Jones et al. [45] conclude that “there is little information or discussion in [the] literature [of health workers behavior and adherence to guidelines] on the drivers of change, the possible mechanisms through which interventions might be acting in order to bring about any observed improvements”. Moreover, Chang et al. [37] warn against over-interpreting positive project outcomes due to the small sample size and suggest that, whilst communication between patients and staff improved upon implementation of the project (as did the quality of care from health workers), the main benefit of the intervention was the ability to make phone calls [37]. Additionally, whilst health workers performance improved, there were no improvements in patient adherence or retention to treatment programs.

### Drug supply chain and stock management

SMS messaging has also been used to improve drug supply chain and management. In Kenya and Tanzania, text messaging has been used as a tool to provide real-time updates on drug stocks in health facilities, reducing out-of-stocks and supporting drug stock management [46,47]. Both pilot projects show that timely data collection on drug stock levels improves availability and supply of drugs to clinics. While these projects show positive results, the lack of RCTs to assess actual impact of mHealth in drug stock management was highlighted [46].

### Disease surveillance and intervention monitoring

The dual burden of rising communicable and non-communicable diseases in Africa, including chronic non-communicable diseases increases, challenges the already over-stretched health systems [48]. Some mHealth projects have targeted disease surveillance and monitoring to reduce disease burden. Selected studies included two pilot projects for malaria reporting [49] and case detection [50], a cross-sectional study to assess malaria control coverage and detection of infections nationwide [51] and a cohort study to improve coverage and scale-up an ART intervention [52]. In Zambia and Uganda, both pilot projects reported on the feasibility of using SMS reporting for malaria active case detection, disease surveillance and case identification [50]. Although timely reporting of data was seen as a positive outcome of the project, the cohort study in Rwanda concluded that mobile phone data collection may be logistically complex and time consuming [52].

### Data collection/transfer and reporting

In many African countries the quality of health information systems tends to be poor due to existent information systems, data incompleteness, untimeliness and inadequate analysis [15]. Mobile phones have been used to tackle these challenges. In Liberia, training was provided to low- and non-literate midwives from rural areas on pregnancy data collection and transmission using mobile phones [53]; authors demonstrate that overall knowledge and skills for data transmission to healthcare data are acquired after the training. Arguably, all the studies included in this review consider some sort of data collection, however, specific consideration was given here to ten projects that assess the collection process as a primary outcome of the research. Five studies were pilot projects [40,54-58], one was an RCT [38], one a mixed methods approach [54], one a cross-sectional study [34] and one an analysis of costs [59]. Use of SMS as a data collection tool was reported as feasible for delivery of information in real time, to improve information quality, reduce data losses and reporting errors, and reduce data uploading difficulties [40,55-58]. However, studies reported several difficulties such as study methodology limitations, privacy and confidentiality matters, low technology use training and skills, no increase of efficiency or reliability of the data, unknown cost-effectiveness, risk of theft, and high implementation costs [56,60].

### Health education and awareness

L’Engle et al. [61] evaluated the provision of automated family planning information to the general public via mobile phones. While the study concludes that it is feasible to use mobile phones for health education and awareness purposes, results showed large underreporting, a risk of bias (e.g. use of contraceptives prior of the implementation of the project) and the need to evaluate impacts using RCTs.

### Results from the SWOT analysis

#### Strengths: what is working?

mHealth projects are highly reliant upon the characteristics of the technology available. Its low-cost, ease of use and wide-spread availability were frequently cited as the main drivers for implementation [18,22,26,43,49,50,56,58],[59,62,63]. An increase in access to mobile phones has motivated researchers and project managers towards seeking innovative ways in which healthcare can be provided, particularly in areas that current infrastructure and technologies cannot possibly reach.

The majority of the projects reported successes and positive outcomes of mHealth in Africa. Some these results were: support to patients in requesting services (e.g. generating appointments) [27,34,62], reduction in communication delays and improvement on data collection and reporting [14,18,38,46,47,49-51,54,56],[63], reduction in patient burden to transportation time and costs [49,51,62], improvement on health workers’ compliance to treatment guidelines [17,22,34,37], increase in patient uptake of counseling and disease testing [22,31,50], and improvement on the patient adherence to treatment [21,22,30-32,37].

Benefits of mHealth projects were described at every level within the healthcare system, from governments to clinic staff, and on to patients. Governments may, for instance, benefit from increased support of patient management [20] and increased direct communication with stakeholders in rural areas [45,50]. Health workers may receive support through professional networks, or can prioritize efforts in areas where they are most needed (e.g. rural areas uncovered by specific programs or interventions) [37,56], and increase their role in active case detection using disease surveillance systems [49]. Finally, patients benefit by saving money from regular consultations, and can also have increased attention and receive more support from health providers, as discussed in previous sections. SMS alone has been proven to help bridge the communication gap in the health sector between health workers and patients, different managerial levels, and between MoH and facilities in the peripheral areas [17,21,22].

A main reasons given for a highly positive perception of mHealth projects by health workers, staff and patients were a high acceptance [16,18,33,34,47,49,56,63] and familiarity of use of mobile phones [31,34,38,47,59,62]. This is understandable given the large reach that mobile phones have had in recent years in Africa. The acceptance of the technology itself may have an effect in the overall acceptance of the project. In Botswana and Uganda for example, the technology was highly accepted and project outcomes were valued positively overall [34,35].

Another characteristic of mobile phones is that it may reduce the feeling of being observed, particularly in situations that might create stigmas. For instance, results show tha patient’s perceived value of the use of mHealth for consultations proved to be more acceptable in sensitive situations where it was more difficult for information to be discussed in face-to-face consultations e.g. HIV-infected patients [62]. The use of mobile phones allowed the patient to keep her privacy.

Other benefits of mHealth are more related to the technology itself. When compared to other technologies (e.g PDAs or laptops), mHealth projects benefited from the fact that mobile phones proved to be less subject to theft and breakage [17,22,25,28,34,54,63].

Although successes have been reported, mHealth integration into the healthcare system is critical to achieve the maximum benefits. Projects have proved to be successful when they have been adapted to the local context and language [31,62], when the government has an existing mHealth or eHealth strategy and has an interest or willingness to set-up a system to integrate mHealth projects [47], and when the project has been developed and implemented by public-private partnerships (e.g. participation of local private service providers) [25,37][47]. Regarding the latter, findings include examples of collaborations between Universities in developed and developing countries, research institutes, non-profit organizations, private sector, public and private hospitals and public sector.

The management of the project is not a simple task and cannot be minimized. Management remains a core component of an mHealth project to ensure that outcomes and goals are achieved. Several factors related to the management and project design were important drivers of project success. Examples include providing adequate incentives (e.g. airtime credit) to ensure a high response rate to the project [21,22] and providing training for staff and users [32,52]. These factors cannot be disregarded by the project manager. Additionally, there are two important factors that characterize management of mHealth projects. Firstly, mHealth projects require minimal human resources and training required is normally simple [32,54]. Secondly, mHealth requires the collection of data during the project implementation, which allows manager to provide real-time supervision and monitor work rate, attendance and working hours of the staff involved [47,50].

Despite the positive conclusions of the studies here presented, it must be noted that the projects are all still small-scale and success of similar large-scale projects is not guaranteed. Fortunately, due to the characteristics of the technology, the estimated low-replication costs and the high adaptability to local cultural settings [51] increasing the potential of these projects for scaling-up [32,47,56], particularly if targeting remote and resource-limited areas [49]. Pilot studies, such as those discussed here, will allow managers to assess impacts prior to scaling-up [27].

On a small-scale, findings from this review show that as accessibility to mobile phones increases, the potential of mHealth to improve healthcare delivery in Africa is also increasing remarkably fast; however, there are weaknesses and challenges that must be considered and addressed for mHealth to fulfill this potential.

#### Weaknesses: what is not working?

One of the major weaknesses of studies on mHealth projects lies in the fact that the claimed benefits are unclear and long-term results remain uncertain [16,32,37,46,61,63]. Often, studies report that cost-effectiveness is unknown [29,61], and in some cases studies mention that the evidence is weak, and the external validity and study design are limited [20,21,27]. The studies report that, while mHealth projects aim to resolve challenges of data collection, during implementation some problems are still faced. These include difficulties in monitoring text message content [23], data under-reporting [37,46], and the possibility of receiving biased responses from participants (e.g. anxiety due to receiving delicate information on the phone) [14,61].

The participation of the government, via the Ministry of Health, is a fundamental aspect for success of mHealth projects. Failure may happen when there is a lack of integration into the healthcare system and, particularly, when there are unclear roles and responsibilities at the various different hierarchical levels (government to managers to health workers) involved in implementation and operation [45]. For example, mHealth outcomes are highly dependent on clinical training, practice and experience of health workers [49,63]. If they are non-existent or provided by the government actors, the project is unlikely to achieve its expected goals.

Project design is also an important task for managers of mHealth projects. In this sense, manager and planners, in particular, have a strong role to play to ensure the success of the project. A major condition of the design is on its adaptability to the local context or tailored to the population’s needs. Projects have an increased risk of failure when they have not been designed for or adapted to the specific context [32].

There are also other important factors related to the project design that need to be looked at closely. For example, planning costs and logistics are essential elements that, if not assessed accordingly, may affect the delivery of the intervention (e.g. timely responses or project coverage) [14,33,61]. Lack of planning may also affect resources available for the implementation. For instance, without adequate planning, there could be occasional shortages of resources, such as staff available, during the project lifespan [22]. This might result in overwhelming staff that have to deal with an increased workload due to the number of messages or calls received [50].

Ease-of-use, familiarity with and access to the technology were important factors mentioned for implementation success. However, in some cases it was reported that, despite the relative ease-of-use, a lack of the skills required to use the technology was a barrier that limited staff in responding and acting promptly to text messaging requests [43,62]. To overcome this problem, some patients asks for support from their relatives or friends, but this may bring other problem particularly when talking patient’s data privacy. In terms of use and acceptability of mobile technology, issues regarding phone ownership such as high phone sharing, lack of money to top-up a phone and male control over household phone ownership may also limit results of a project [32,37].

The capacity of mHealth projects is defined by the capacity of the technology itself. There are for instance, only a limited number of characters that can be sent using text message [18], thus limiting the application of these projects to specific types of interventions. Thus, the use other technologies to replicate or imitate the same mHealth project may not result in similar outcomes [21,25,54]. Other technology-related problems are poor data quality and transfer [18,37], network loses, phone maintenance costs [17], risk of theft and loss [19], poor handling and use [57], a lack of software flexibility and adaptability, and risk of human errors in the program [63].

In addition to these technical challenges, legal issues arise in terms of privacy and security measures to be taken for obtaining, handling and transmitting data. As a new concept that has gained popularity in recent years (most of the studies are from 2009 onwards), little was found mentioned in terms of legal factors, standards and regulations surrounding the use and application of mHealth for healthcare services. For instance, privacy concerns exist when a patient is not the direct owner of the mobile phone [34,56]. In this sense, two possible solutions were found in the results: firstly, the use of coded information for confidentiality protection and data security [63], and the integration of SMS guidelines into broader clinical and healthcare processes [50]. Though during project implementation some measures were taken by researches (e.g. a security PIN code to protect patient data), these concepts remain highly unfamiliar and very poorly understood by users [37,54]. Moreover, user expectations in terms of confidentiality and privacy remain very variable even within the same population [32].

#### Threats: why is it not working?

In Africa, mHealth projects may face external barriers and limitations that might cause project’s failure. Limiting factors need to be considered from early phases of the project. So far, it was discussed that the project design and the adaptation to the local context were critical issues that, if not thoroughly assessed, could result, worst case scenario, in a complete project failure. These are factors internal to the project, and can be controlled by the project manager, researcher or planner.

The limited research and knowledge available on mobile phone applications in health [22], and the scarce knowledge of the long-term effect of mHealth intervention on health outcomes in low-resource settings [15,62] are two external factors that may limit potential impacts of mHealth interventions. There are other factors that may also threaten project sustainability and delivery of results. These include the limited funding opportunities available for long-term implementations [56] and the dependency of external factors unrelated to the project itself, such as the duration of patient treatment that may influence adherence and motivation to participate [18,63], user’s illiteracy and high workload for facility staff or health workers [38,54].

Some of the threats for mHealth lie in the health system and government themselves. The role and level of involvement of governmental organizations is fundamental for project success during its lifespan. However, for the integration of mHealth into the current systems, care delivery processes as they stand today need to be redesigned (e.g. change to electronic records and data) [22]. Healthcare providers need to develop the cultural and organizational capacity required to manage digital health information [63]. The lack of these capacities may lead to late reporting, lack of feedback and incomplete data collection [63]. Mechanisms to use data collected are also required at the district and national levels [17,32,43,46,56]. Evidence shows that there are wide gaps in the understanding of the complexities that health systems may face in large scale implementations of mHealth projects [55], or the standards required for monitoring and evaluating them [21,34]. A lack of guidance and policies from the Ministry of Health and inexistent financial support from governments to deploy mHealth projects are regarded as reasons for failure [49].

Probably the major limitation for implementation of mHealth projects is the coverage and accessibility of the technologies. mHealth is highly dependent on infrastructure availability in the area where the project is being deployed, hence a reliable network, internet and electricity access [19,32,34,37,38] are prerequisites. Access to mobile phones in Africa is extensive, but not necessarily reliable. Moreover, the technical or expert knowledge for maintenance and development of platforms (software and hardware) may be limited or not available locally [37,56], and when available, the lack of expertise may slowdown implementation as technical training will normally be required [49,57,62].

It was previously mention that acceptance was a reason for the success of mHealth project. However, even if a pilot project may have been perceived as valuable by the user, there are still questions regarding acceptance of the technology by the communities as receptiveness is limited by socioeconomic and sociocultural factors, geographic barriers and quality of care [19,20,32,33,49]. For instance, in some places SMS interventions may fail due to high illiteracy levels and/or user preferences for making voice calls or personal appointments [14,17,62]. In addition to all these factors, little is known in regards of data protection, and the feasibility and acceptability confidentiality methods [62].

Due to all the external factors that may threaten success of mHealth project, it is important to point out the relevance of the role that management has before, during and after implementation. The unknown cost-effectiveness of deployment and maintenance [49,56,63], particularly on a large-scale [32], and the challenges of management of mHealth projects remain underestimated [26]. As we gain more knowledge on mHealth interventions, opportunities need to be carefully assessed for further implementations.

#### Opportunities and recommendations

Opportunities to increase implementation and expand applications of mHealth in Africa are vast, but future steps need to be taken cautiously. Tomlinson *et al.*[64] have recently published a review where they discuss the lack of evidence regarding mHealth effectiveness and efficacy [64]. The authors argue that an mHealth project is likely to work if there is a follow-up of the project, if it has been designed for specific contexts, and if high consideration has been given to the frequency of the message delivery, wording and content of the message. While mHealth projects face challenges and threats, there are also many opportunities. The major opportunity for mHealth is the increasing mobile phone coverage. As coverage to mobile phone networks expands and new communication technologies are developed (e.g. cheaper smartphones), opportunities for mHealth applications remain high [14-19,22,26,28,31,34,37,40],[43,49,56,62]. For instance, innovations for automated text messaging and partnerships with mobile technology developers may improve scalability [37,39,49]

However, large-scale or nationwide coverage of mHealth projects were rarely reported in the literature. While, potential for scaling them up was frequently mentioned [32,47,56], the lack of secure funds for scaling-up [19,32], the potentially high set-up costs and unknown cost-effectiveness [43,63], and the lack of evidence of effectiveness to assess, for instance, the applicability of mHealth to other diseases [24,40,44,54,62] are factors that just allow small scales and limited interventions.

Solutions for the challenges faced by the health systems in Africa are very much needed. As previously discussed, opportunities are high where the application of mHealth can support drug supply and stock management [43], stock forecasting [15,47,49], collect and disseminate health data in remote areas [33,58], support different national disease control programmes and provide access to data for an evidence-based approach [47,50], utilize health workforce (e.g. health workers) [19,47,49] and provide specialized care/mentoring in rural areas [26,50]. Considering that these projects may be highly attractive and acceptable for private or commercial partners and governments (MoH) [21,29,49,50], future projects should consider larger-scales and the full integration into the healthcare systems.

**Figure 2:**
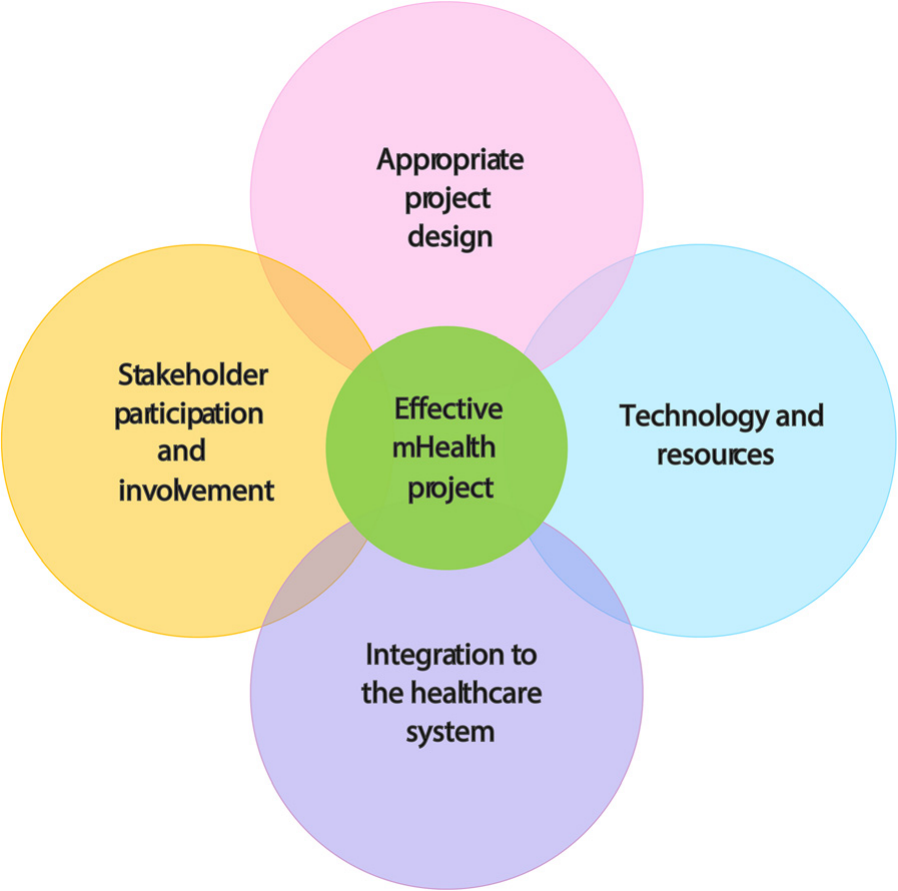
**Main considerations for an effective mHealth project in the African context.** Good project design (adapted to the local context, promotion, education and awareness of the project, etc.), Technology and resources (use local resources, capacity building, availability and maintenance), Involvement of stakeholders (strong public-private partnership, multidisciplinary teams, MoH, political leadership, local champion) and Government e-health/m-health department (program monitoring and evaluation, research, etc.).

Recent research into health outcomes of mHealth interventions have been increasing rapidly. According to the findings of this study, the major threats to mHealth projects include cultural perception, language, limited resources in rural settings, weak health systems and external financing schemes. Whilst factors may be setting-dependent, Figure 2 provides an overview of the fundamental elements identified that could lead mHealth projects to succeed in Africa and integrates the findings of this study into a tabulated SWOT analysis.

### Implications and limitations of the study

A major strength from the present review is that, to the knowledge of the authors, it is the first time that a systematic review has been performed to understand and inform on aspects surrounding mHealth project implementations in poor settings in general. The results from this study are consistent with the existing literature, advocating for mobile phone technologies as useful tools for health interventions seeking to improve health outcomes in developing countries [17,21,22,43,60]. Results show that more evidence-based research is needed in the field of mHealth implementations, especially for large-scale and longer-term implementations. Pilot studies allow to test feasibility at small-scales, and but the potential of mHealth has not been fully explored–though frequently mentioned.

However, this study presents some limitations. Firstly, studies included were publications in English, limiting findings of projects published in French and Spanish. Secondly, we decided to include only published peer-reviewed literature. The grey literature contains a vast amount of rich experiences on mHealth project implementations in Africa. While we regard them as valuable, through the present systematic review we have attempted to compile theory objective according to the evidence available. While part of our knowledge on the topic has come from reading grey literature, we consider that a large proportion of them tend to present positive results only. This is because many of these projects have been support with funding from major donors sources, which demand results and effective outcomes. These demands put pressure on managers who end up reporting only on the positives, leaving the negatives in the obscurity. Since we aimed to formulate conclusions with a high level of rigor, we decided to narrow our scope to peer-reviewed literature. We admit that this is certainly the main limitation of the present study.

## Conclusions

The results from this study on mHealth projects in Africa sought to answer, *“what is working with regards to improving population health? what is not working, and why?”* mHealth implementations pose a potential to become an important part of the health sector to establishing innovative approaches to delivering care and benefits have been highly praised, but is clear that mHealth projects are not a solution to the challenges that health systems face in many African countries. Evidence remains poor, results are still project- or setting-specific and questions regarding impact, scalability, increase coverage (e.g. different diseases, different settings, different target populations), cost-effectiveness and sustainability of the projects in Africa are yet to be addressed. While mobile phone technology continues to improve, more research on these areas is essential to fully understand the potential of these projects and help to reach the hard isolated and marginalized communities in low and middle income countries (LMICs).

## Competing interests

The author(s) declare that they have no competing interests.

## Authors’ contributions

NMD contributed to the conception and design of the study, the acquisition, analysis and interpretation of the data, the drafting and finalization of the manuscript. CAJ contributed to acquisition, analysis and interpretation of the data, drafting, revision, finalization and final approval of the version to be published. SL contributed to the concept and design of the study, analysis and interpretation of the data, drafting and revision of the manuscript and final approval of the version to be published. All authors read and approved the final manuscript.

## Pre-publication history

The pre-publication history for this paper can be accessed here:

http://www.biomedcentral.com/1471-2458/14/188/prepub
